# Improving survival in Duchenne muscular dystrophy across eras: a systematic review and cumulative meta-analysis

**DOI:** 10.1007/s00415-026-14121-4

**Published:** 2026-09-06

**Authors:** Lara Benning, Zoe Bousraou, Bastian Gruber, Silvia Ulrich, Esther I. Schwarz

**Affiliations:** 1 Sleep Disorders Centre, Department of Pulmonology, University Hospital Zurich, Raemistrasse 100, 8091 Zurich, Switzerland; 2 University of Zurich, Raemistrasse 71, 8006 Zurich, Switzerland

**Keywords:** Duchenne muscular dystrophy, Home mechanical ventilation, Cardiomyopathy, Survival, Life expectancy, Mortality

## Abstract

**Background:**

Duchenne muscular dystrophy (DMD) was historically associated with death in the late teens or early twenties, mainly from respiratory failure. Survival has improved substantially with home mechanical ventilation (HMV) and multidisciplinary care, although variability remains. This study evaluated temporal trends in survival in DMD and the impact of HMV.

**Methods:**

A study-level cumulative meta-analysis (PROSPERO CRD420251163011) of studies reporting survival outcomes in patients with DMD was conducted (PubMed 1977 to 13 October 2025). Pooled estimates of median survival were calculated, and random-effects meta-analyses with predefined subgroups (HMV and study period) were performed, alongside meta-regressions. Risk of bias was assessed using the Newcastle–Ottawa Scale.

**Results:**

53 studies (median follow-up 8 years), comprising more than 13,000 patients, of whom 60% received HMV, were included. Median survival differed substantially between ventilated (29 years, 95%CI 27 to 31) and non-ventilated (19 years, 95%CI 18 to 20) patients. Survival improved progressively over time in both groups. Glucocorticoid therapy was not associated with improved survival (p=0.45), whereas treatment with heart failure medications, including renin-angiotensin system inhibitors (p=0.002) and β-blockers (p=0.02), was associated with longer survival. The predominance of mortality shifted from respiratory to cardiac causes, while enhanced cardiac management was associated with a growing contribution of other causes of death.

**Conclusion:**

Survival in DMD has increased substantially over time, with median survival now approaching the third decade of life among ventilated patients. The growing contribution of cardiac and other non-respiratory causes of death highlights the importance of long-term multidisciplinary and early cardioprotective intervention.

**Study registration:**

The meta-analysis and systematic review have been registered on PROSPERO (CRD420251163011).

**Supplementary Information:**

The online version contains supplementary material available at https://doi.org/10.1007/s00415-026-14121-4.

## Introduction

Duchenne muscular dystrophy (DMD) is the most common inherited neuromuscular disorder associated with progressive respiratory muscle involvement. DMD is caused by mutations in the dystrophin gene leading to the absence of functional dystrophin protein [1]. Loss of ambulation typically occurs in early adolescence due to progressive skeletal muscle weakness, followed by cardiomyopathy and hypercapnic respiratory failure that significantly impact morbidity and mortality [2]. Historically, the prognosis of DMD was poor, with most patients dying in their late teens or early twenties, predominantly as a consequence of respiratory failure. A major shift in the natural history of the disease occurred with the introduction of long-term invasive ventilation (IV) via tracheostomy, which significantly prolonged survival [3]. However, it was the subsequent adoption of non-invasive home mechanical ventilation (NIV, HMV) delivered via a nasal or oro-nasal (facial) mask that marked a transformative step in care [4]. NIV offered effective ventilatory support with fewer complications, improved quality of life, and better integration into home care settings. Its widespread use has led to a further increase in survival, with many patients now living into their late twenties or thirties, and some into their forties and beyond, particularly in settings with access to comprehensive multidisciplinary care [5–7]. Whereas respiratory failure was previously the predominant cause of mortality, cardiac disease has emerged as the leading cause of death in contemporary cohorts [8, 9]. Additional contributors to mortality include lower respiratory tract infections, gastrointestinal complications, and adverse events related to anaesthesia and surgical procedures [10–12]. Today, the management of DMD requires proactive and integrated respiratory and cardiac monitoring. The role of corticosteroids after the loss of ambulation and evolving genetic therapies remains unclear [13]. Despite these advances, marked disparities in survival and clinical outcomes persist, probably reflecting differences in access to specialised care, socioeconomic factors, and regional variations in healthcare delivery. These inequities highlight the continuing need for equitable access to comprehensive, multidisciplinary management strategies for individuals with DMD.

A systematic meta-analysis and review on survival in DMD was performed to systematically assess how life expectancy has changed over time. By comparing historical cohorts with more recent data, this study assesses the impact of HMV and heart failure therapies on survival and evaluates potential persistent disparities. The aim of this study was to provide insight into global survival trends and to inform future clinical guidelines that promote evidence-based strategies for improving outcomes in individuals with DMD worldwide.

## Methods

### Study registration and data sources

A study-level meta-analysis was performed in accordance with the ‘Meta-analyses Of Observational Studies in Epidemiology (MOOSE)’ Checklist [14] and was previously registered in PROSPERO (CRD420251163011). A systematic literature search on PubMed (MEDLINE) database was conducted (November 1977 to June 30, 2026), and references of existing meta-analyses and reviews on survival in DMD were screened. Details on the literature search are provided in the Online Resource.

### Study eligibility and data extraction

Duplicates were removed, and studies were screened for eligibility based on titles and abstracts. Full-text articles deemed potentially eligible were independently reviewed by two authors (LB, ZB). Inclusion criteria were 1) individuals with DMD, 2) reporting appropriate survival data (median survival or age at death), 3) observational or interventional study design, and 4) article in English. Studies including neuromuscular disorders other than DMD were excluded. If data on one cohort was published more than once, the earliest and most comprehensive dataset was used. Data from included studies were extracted into a spreadsheet recording study details (study design, publication year, inclusion period, country, continent, intervention, number of patients, follow-up time), patient anthropometrics (birth cohort, body mass index (BMI)), medications (heart failure drugs, glucocorticoids), ventilation status (HMV % total, NIV, IV), and survival data (median survival, age at death, % deceased, cause of death). Where studies reported different birth cohorts or interventions, these were extracted and analysed as separate subgroups. Missing data were recorded as unavailable.

### Risk-of-bias assessment

The Newcastle–Ottawa scale (NOS) for cohort studies was used to assess the risk of bias [15]. Two authors (LB, ZB) estimated the risk-of-bias for each publication. For instance, risk-of-bias arose when the stated follow-up interval was not sufficient to address survival or when the follow-up period was not reported. 0 to 3 stars were considered as high risk, 4 to 6 stars as moderate risk, and 7 to 9 stars as low risk. Disagreements were discussed and resolved by consensus.

### Outcomes

The main outcomes of interest were the median survival in DMD patients overall, survival stratified by period (⪅1980, ⪅1990, ⪅2000, ⪅2010, ⪅2020), and survival stratified by ventilation status (no HMV vs HMV). Additional outcomes included the association of median survival with drug therapies (glucocorticoids, heart failure medications such as angiotensin-converting enzyme inhibitors (ACEI) and/or angiotensin receptor blockers (ARB) and beta-blockers), as well as the age at death and the causes of death per period. Studies were allocated to specific periods by either (in descending order of relevance) year of birth, year of study inclusion, or year of publication.

### Statistical analysis

Normality of variable distributions was assessed using quartile-quantile plots and Shapiro–Wilk test. Baseline characteristics are presented as median (IQR) or number (%). Median survival values were summarised overall and across different time periods. A study-level random-effects meta-analysis was performed, with studies weighted by inverse variance. Between-study heterogeneity was estimated using restricted maximum likelihood (REML), and confidence intervals were adjusted using the Hartung–Knapp method. Heterogeneity was assessed using the Q test and I^2^ statistic. For studies presenting Kaplan–Meier curves without reporting median survival, median survival was estimated using the method described by Broomfield et al. [16]. A median meta-analysis was constrained by the limited reporting of interquartile ranges (IQR) or 95% confidence intervals (CI) in the majority of studies. Consequently, we conducted a conventional ‘meta-analysis of means’ assuming equivalence between the median and the mean after verifying negligible skewness. Standard errors (SE) were derived from studies reporting IQR by $\mathrm{S}\mathrm{E} \approx \frac{\mathrm{q}3-\mathrm{q}1}{1.35 \mathrm{x} \sqrt{\mathrm{n}}}$ [17], from 95% CI by $\mathrm{S}\mathrm{E} =\frac{\mathrm{u}\mathrm{p}\mathrm{p}\mathrm{e}\mathrm{r} \mathrm{l}\mathrm{i}\mathrm{m}\mathrm{i}\mathrm{t} - \mathrm{l}\mathrm{o}\mathrm{w}\mathrm{e}\mathrm{r} \mathrm{l}\mathrm{i}\mathrm{m}\mathrm{i}\mathrm{t}}{3.92}$ [18] or imputed as a sample-size-weighted median SE derived from available SE (${\mathrm{S}\mathrm{E}}_{imputed} \approx {\mathrm{S}\mathrm{E}}_{median} \mathrm{x} \surd \frac{{\mathrm{n}}_{median}}{{n}_{imputed}}$) [19]. Studies were stratified to their ventilation status (studies with or without ventilated patients), and conventional and cumulative meta-analyses were performed. Meta-regressions (mixed effects models) were run with clinically relevant variables with sufficient reporting. For regression models with multiple predictors, Pearson correlation and multicollinearity were assessed. For the age at death analysis, median values were transformed into means for studies that reported medians only [17]. Sensitivity analyses were performed regarding risk-of-bias with risk category as a moderator variable in meta-regression and alternative period ordering. Possible publication bias was assessed using funnel plots and Egger’s regression test. Statistical significance was set at α < 0.05, with effects reported at 95% CI. RStudio (version 2025.9.2.418; package: meta, functions: metagen, metacum) was used for all analyses and figures.

## Results

### Included studies and patients

**Fig. 1:**
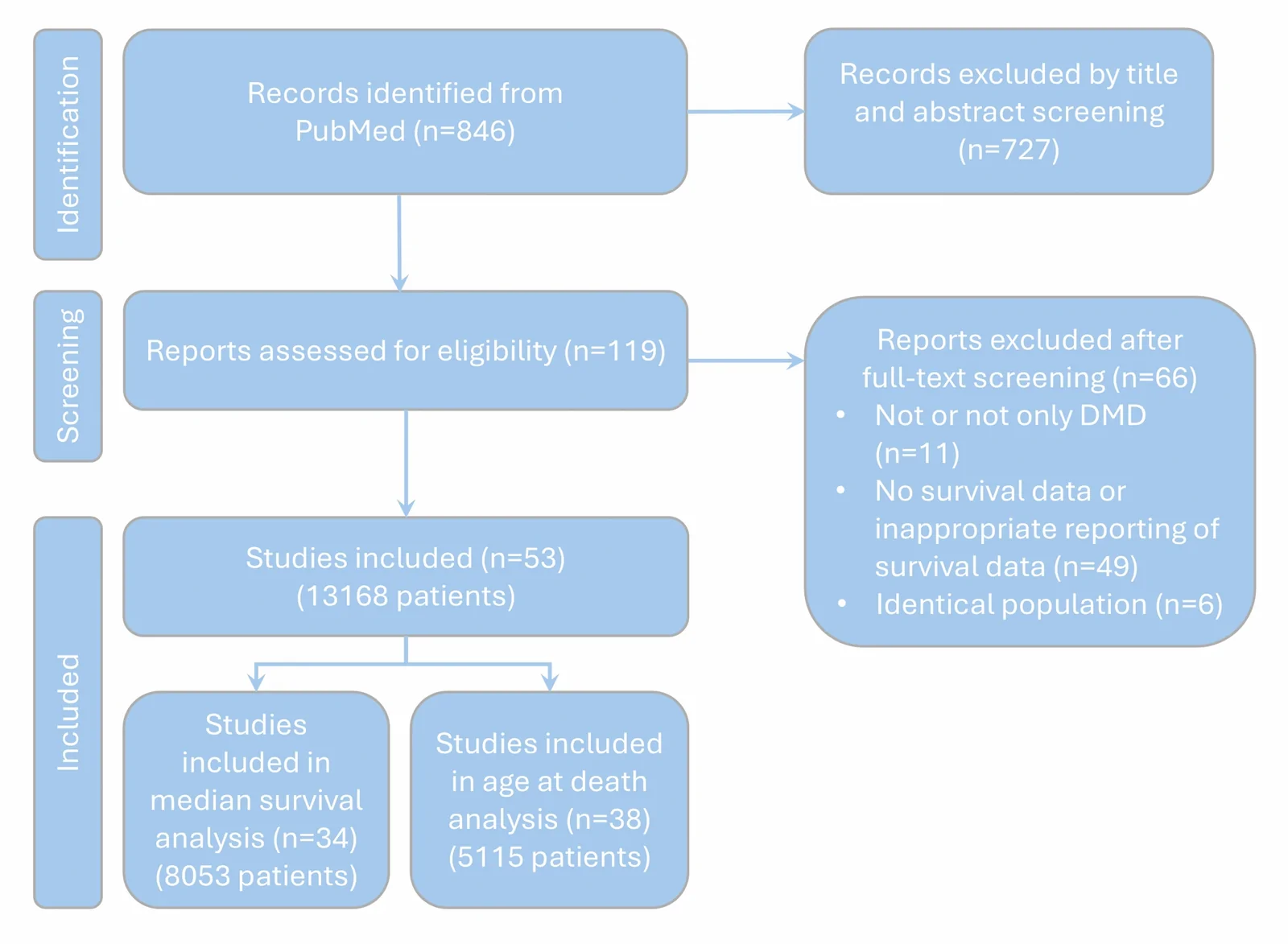
Study flow A total of 53 studies (13′168 patients) were included (34 studies in the median survival analysis and 38 studies in the age-at-death analysis)

A total of 53 studies (44 retrospective and 9 prospective), comprising more than 13,000 patients with DMD, were included in the final analysis [3, 8, 20–70]. Of these, 34 studies reported median survival, while 38 reported age at death. Most studies were conducted in Europe (51%), North America (28%), and Asia (15%), with a median follow-up duration of 8.2 (6.6–11.1) years. The median age of patients was 14.2 (6.3–19.9) years, and the median BMI was 21.8 (20.2–22.9) kg/m^2^. Overall, 41.4% of patients were deceased at the time of reporting. A median of 59.5 (0–100) % of patients received HMV, while 32.7 (14.7–68.9) % used mechanical in-/exsufflation (cough assist device) (Fig. 1, Table 1).

**Table 1:** Patient anthropometrics and study characteristics for studies reporting on median survival and/or age at death

Variable	Median (IQR)/n (%)
*Patient anthropometrics*	
Age (years)	n=50, 14.2 (6.3–19.9)
BMI (kg/m^2^)	n=9, 21.8 (20.2–22.9)
Deceased (%) (overall)	n=66, 41.4 (24.2–63.5)
in median survival analysis	n=36, 45.8 (33.7–63.8)
in age at death analysis	n=51, 38 (19.2–58.7)
Home mechanical ventilation (%)	n=60, 59.5 (0–100)
- Non-invasive ventilation (%)	n=24, 77.1 (9.6–100) (of studies with HMV) n=39, 1.2 (0–78.9) (of all studies)
- Invasive ventilation (%)	n=24, 14 (0–34.7) (of studies with HMV) n=37, 0 (0–21.7) (of all studies)
Cough assist (%)	n=7, 32.7 (14.7–68.9)
*Medication*	
- Glucocorticoids (%)	n=41, 35.3 (16.2–64.4)
- *Heart failure medication (%)*	
ACEI or ARB	n=19, 77.1 (49.4–89.3)
Beta-blocker	n=15, 31 (16–45.4)
Aldosterone antagonists	n=9, 9 (4.4–9.1)
Diuretics	n=7, 20 (8.4–35.8)
Glycosides	n=8, 16.5 (6–36.3)
*Study characteristics*	
*Study design*	
- Retrospective	44 (83)
- Prospective	9 (17)
*Continent*	
- Asia	8 (15)
- Africa	0 (0)
- Australia	2 (4)
- Europe	27 (51)
- North America	15 (28)
- South America	1 (2)
Average number of patients per study	n=101, 57 (27–137) (in total: 13,168, for median survival: 8053, for age at death: 5115)
Follow-up time (years)	n=38, 8.2 (6.6–11.1)

*ACEI* Angiotensin-converting enzyme inhibitor, *ARB* Angiotensin receptor blocker. Number (n) of studies reporting on variable

### Median survival by period, ventilation status, and associated factors

**Fig. 2:**
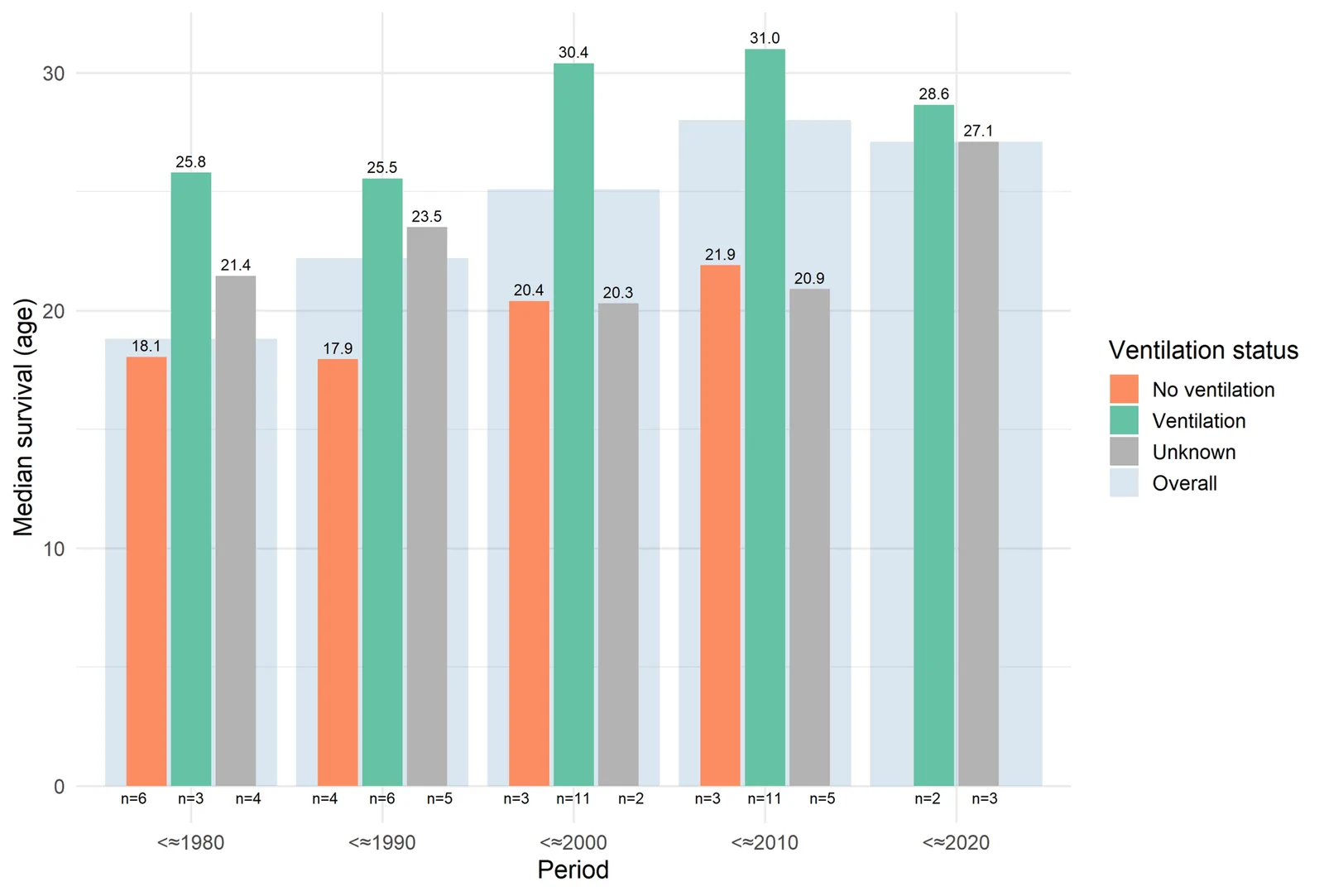
Median survival (years) by study period and ventilation status The number of studies is shown below each bar. Overall, there is an upward trajectory regarding median survival over the different periods (18.8 years in the period ⪅1980 to 28 and 27.1 years in the periods ⪅2010 and ⪅2020, respectively) with studies with ventilated patients having a higher life expectancy than those without

Overall, median survival was 23.9 (20.3–29.9) years. Over the past decades, median survival increased steadily from 18.8 (18–22.7, n=13) years in the period ⪅1980 to 22.2 (20.4–26.8, n= 15) and 25.1 (21.2–31.3, n=16) years in the periods ⪅1990 and ⪅2000, respectively, and further to 28 (22–32.9, n=19) and 27.1 (26.5–28.4, n=5) years in the periods ⪅2010 and ⪅2020, respectively. Median survival differed substantially between studies that included ventilated patients and those that did not. However, both groups demonstrated a similar upward temporal trend in survival (Fig. 2).

**Fig. 3:**
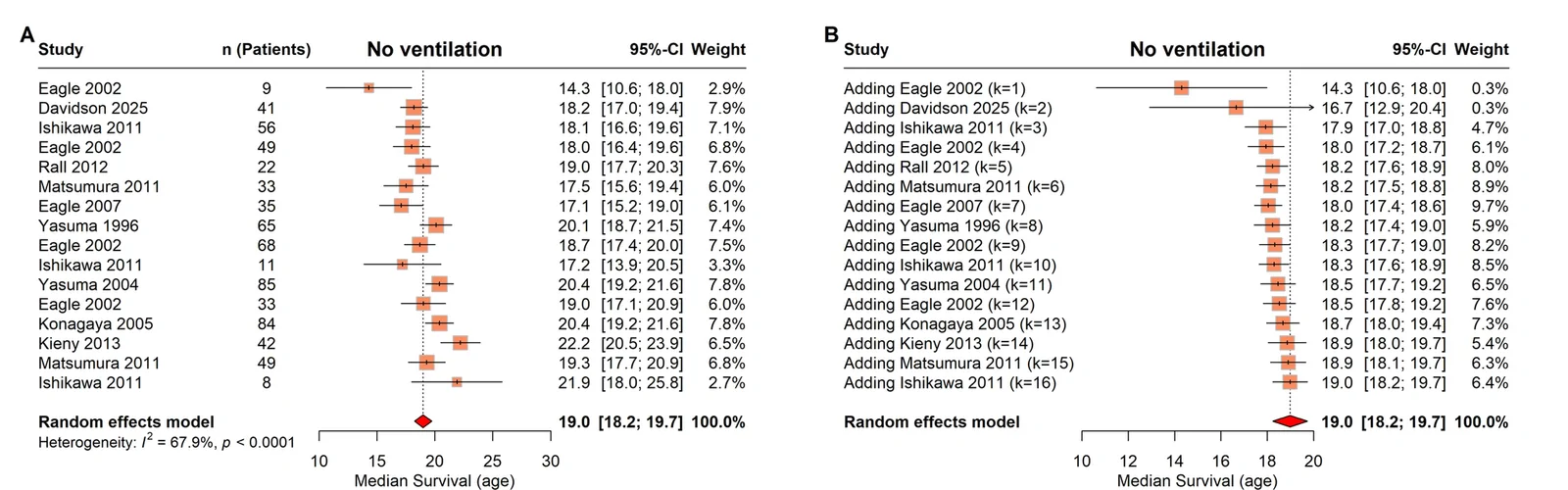
Meta-analysis and forest plot of median survival in studies including non-ventilated patients Meta-analysis demonstrates a median survival of 19 years in studies including non-ventilated patients. Cumulative meta-analysis shows a slightly improved survival with time. **a** Conventional meta-analysis, **b** Cumulative meta-analysis

**Fig. 4:**
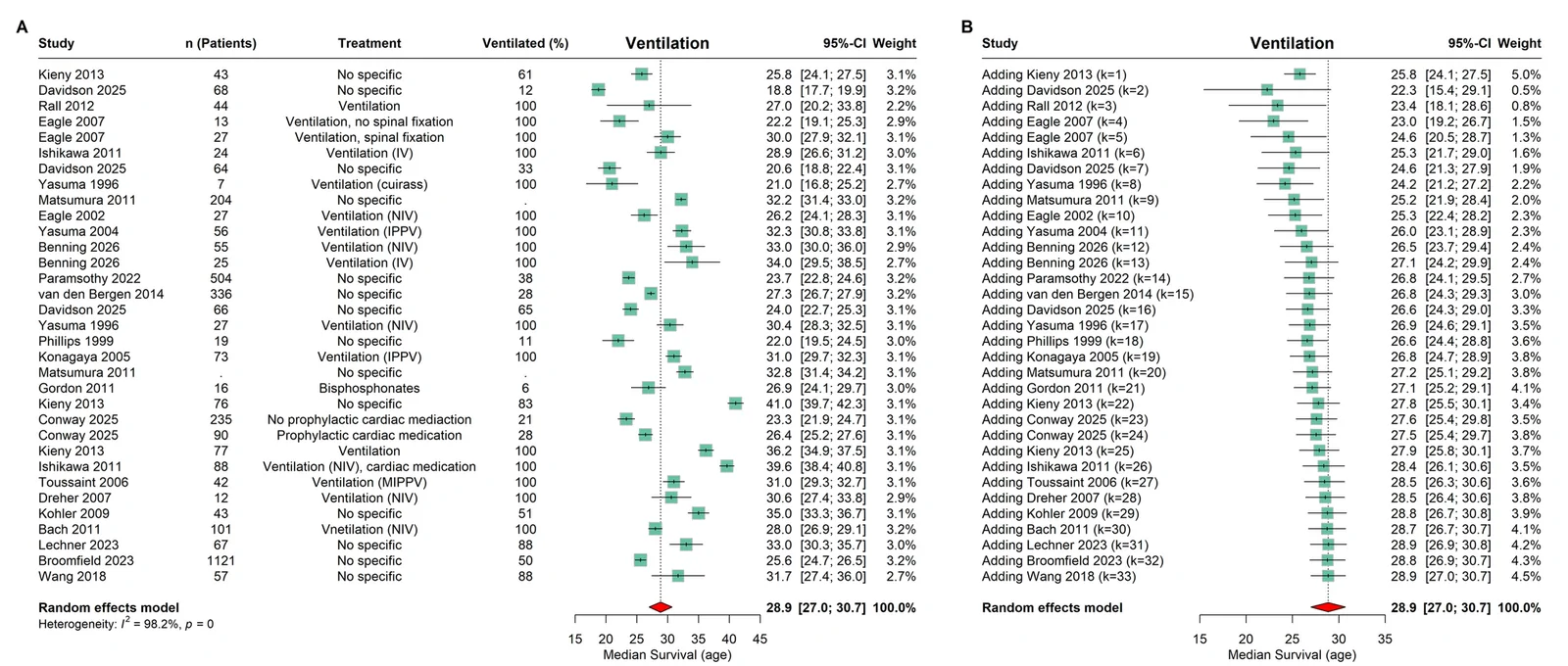
Meta-analysis and forest plot of median survival in studies including ventilated patients Meta-analysis demonstrates a median survival of around 29 years in studies including ventilated patients. The proportion of ventilated patients per study differs. Cumulative meta-analysis shows a slightly improved survival with time. **a** Conventional meta-analysis, **b** Cumulative meta-analysis. *IPPV* intermittent positive-pressure ventilation, *IV* invasive ventilation, *MIPPV* mouthpiece intermittent positive-pressure ventilation, *NIV* non-invasive ventilation

Meta-analysis of studies that did not include ventilated patients yielded a pooled median survival of 19.0 years (95% CI 18.2 to19.7; n=16), with significant between-study heterogeneity (Q test p<0.0001; I^2^=67.9%). Cumulative meta-analysis showed a progressive increase in median survival over time (Fig. 3A, B). Among studies that included ventilated patients, the pooled median survival was 28.9 years (95% CI 27.0 to30.7; n=33), with substantial heterogeneity (Q test p<0.0001; I^2^=98.2%). Cumulative meta-analysis likewise demonstrated a sustained improvement in survival over time (Fig. 4A, B).

**Fig. 5:**
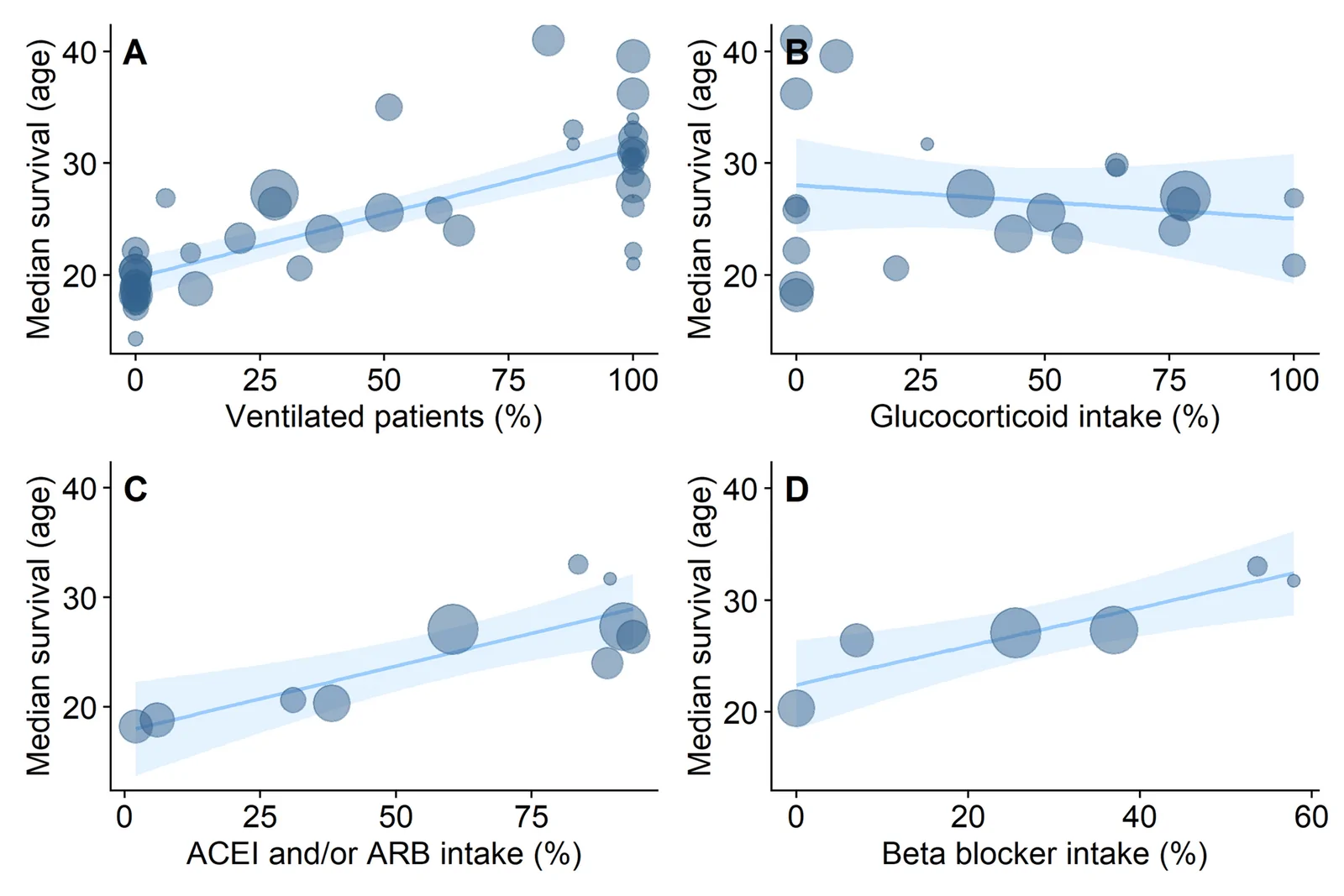
Meta-regression and bubble plots of median survival according to treatment characteristics **a** Proportion of ventilated patients, **b** Proportion receiving glucocorticoids, **c** Proportion receiving angiotensin converting enzyme inhibitors (ACEI) and/or angiotensin receptor blockers (ARB), **d** Proportion receiving beta-blockers. The bubble size is inversely proportional to the standard error. Larger bubbles represent studies with smaller standard error and higher impact

Meta-regression demonstrated that a higher proportion of ventilated patients within studies was significantly associated with longer median survival (n=47, p<0.0001, β1=0.12, 95% CI 0.09 to 0.14, R^2^=63.6%; Fig. 5A). When comparing NIV (n=28, p<0.0001, β1=0.12, 95% CI 0.08 to 0.16, R^2^=74.4%) to IV (n=28, p<0.0001, β1=0.17, 95% CI 0.12 to 0.23, R^2^=74.4%) utilisation, there was no significant difference in survival between the two ventilation modalities (p=0.1; correlation between modalities r=− 0.23, VIF= 1.03).

Glucocorticoid use was not associated with survival (n=21, p=0.45, β1=−0.03, 95% CI −0.11 to 0.05, R^2^=0%; Fig. 5B). After adjusting for the period, the analysis stayed non-significant (n=21, p=0.06, β1=−0.07, 95% CI −0.15 to 0.004, R2=28.5%). In contrast, the use of heart failure therapies was associated with significantly improved survival, including angiotensin-converting enzyme inhibitors (ACEIs) and/or angiotensin receptor blockers (ARBs) (n=10, p=0.002, β1=0.11, 95% CI 0.05 to 0.18, R^2^=70.3%; Fig. 5C) and beta-blockers (n=6, p=0.02, β1=−0.17, 95% CI 0.06 to 0.29, R^2^=77.9%; Fig. 5D), However, the beta-blocker analysis was based on a limited number of studies (6 studies). All findings remained consistent after adjustment for study period.

### Median age at death and cause of death per period

The overall median age at death was 21.9 (19.5–26.7) years, with period-specific medians of 18.6 (18.5–18.9, n=5), 20 (29.5–25.8, n=7), 20.4 (19.1–25.3, n=18), 25.1 (21.2–29.5, n=16), and 23 (20.3–26.8, n=20) years for ⪅1980, ⪅1990, ⪅2000, ⪅2010, and ⪅2020, respectively.

**Fig. 6:**
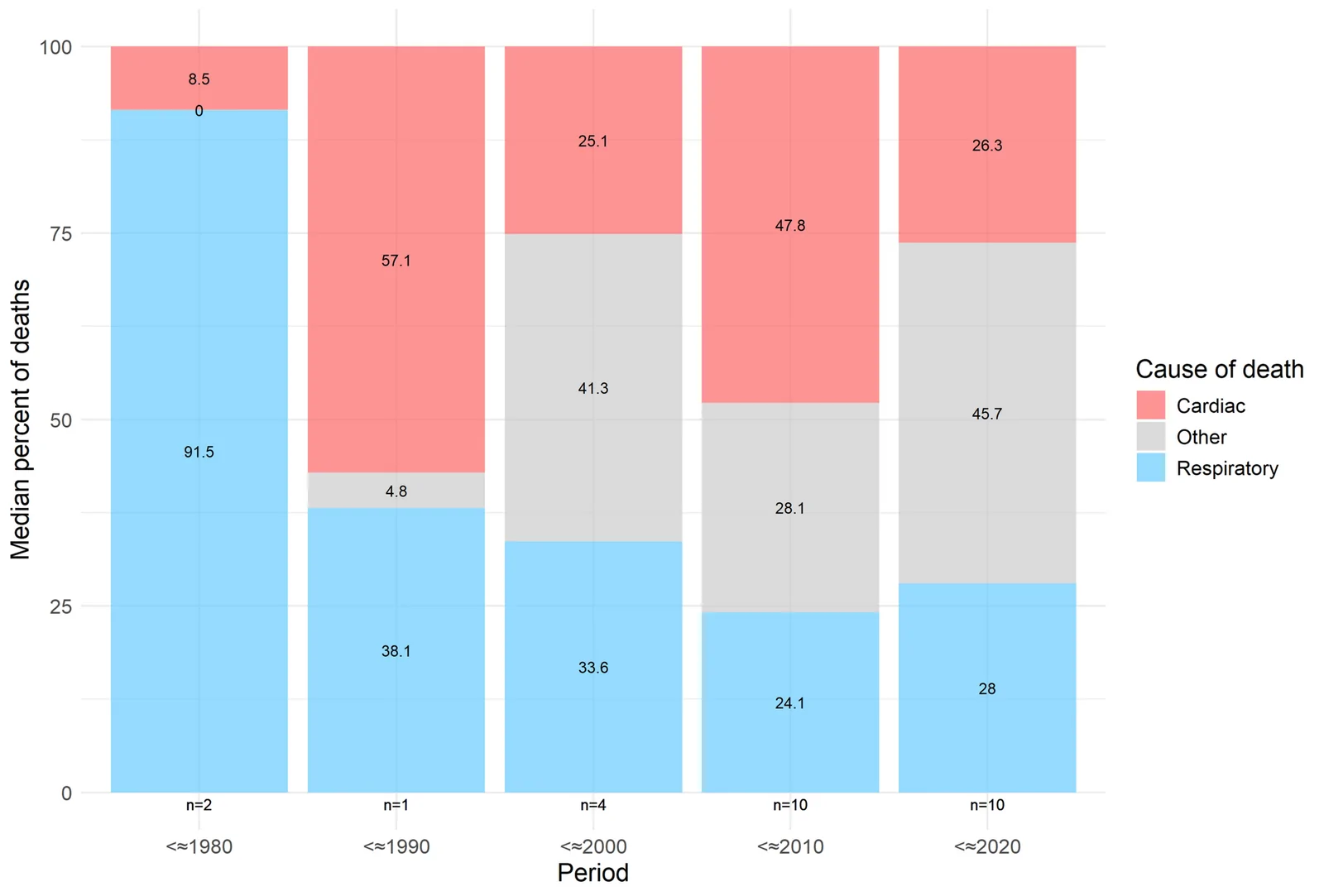
Causes of death (respiratory, cardiac, and other) by study period. Respiratory causes have a large proportion in earlier periods (around 92% in ⪅1980). Over time, cardiac causes and other causes replace the respiratory ones

In earlier periods, death was predominantly due to respiratory causes (91.5% in ≤1980). Cardiac causes became increasingly prominent in the subsequent period (57.1% in ≤1990), while in more recent periods other causes have emerged (45.7% in ≤2020) (Fig. 6).

### Sensitivity analysis, risk-of-bias, and publication bias

As period classification was not fully consistent across all studies, sensitivity analyses using alternative period orderings were performed, with all results remaining robust. Of the studies reporting median survival, 25 were rated as low risk of bias, 43 as moderate risk, and none as high risk (**Online Resource S1**). Meta-regression using risk-of-bias category as a moderator showed no significant effect (p=0.87). Funnel plots and Egger’s regression tests revealed no evidence of publication bias in studies including (p=0.93) or not including ventilated patients (p=0.29) (Online Resource S1).

## Discussion

In this cumulative meta-analysis of more than 13,000 patients across 53 studies, we demonstrate a progressive increase in median survival over time from 19 to 29 years, with a clear survival advantage in cohorts including ventilated patients. However, marked between-study heterogeneity persists, and survival continues to vary according to management strategies. In parallel with increasing longevity, the pattern of mortality has shifted from predominantly respiratory causes in earlier decades towards a growing contribution of cardiac and other non-respiratory causes in more recent periods, highlighting the evolving natural history of DMD in the modern treatment era.

There are only two other meta-analyses on survival in DMD, each including approximately one third of available patients and using differing methodological approaches; nevertheless, overall median survival estimates are broadly consistent with our findings [7, 16]. In this largest and most recent meta-analysis of survival in patients with DMD, median survival differed substantially between ventilated (29 years) and non-ventilated (19 years) patients, with both groups demonstrating a progressive improvement over time. This analysis indicates that ventilation modality (non-invasive versus invasive) does not significantly influence median survival, consistent with previous reports [23, 70]. Beyond survival outcomes, patient-reported satisfaction and quality of life are generally high, with over 80% of ventilated patients expressing satisfaction irrespective of ventilation modality [6]. Patients receiving HMV and gastrostomy feeding are at increased risk of acute gastrointestinal complications [71]. While continuous NIV combined with mechanical in-/exsufflation may prolong survival and delay tracheotomy [21], switching from NIV to IV is appropriate when NIV becomes ineffective. However, primary invasive ventilation has been associated with higher rates of acute respiratory failure and mortality [23, 70].

Although guidelines recommend initiating HMV, preferably nocturnal NIV, based on sleep studies and pulmonary function tests [72], no standardised HMV initiation criteria exist. Nocturnal HMV is typically initiated around the age of 20 years, with forced vital capacity (FVC) as a key predictor of nocturnal hypoventilation, and an extension to daytime ventilation may become necessary in the mid-twenties [70]. An FVC below 1 Liter is a strong negative predictor of subsequent mortality in DMD [51]. Initiation of HMV slows the decline in lung function and respiratory muscle strength [70]. Both the distribution of causes of death and age at death have shifted upwards in respiratory and cardiac death [73]. Respiratory causes have been largely superseded by cardiac causes, while with improved attention to cardiac protection, other causes of death like gastrointestinal complications seem on the rise. Progressive cardiomyopathy is a major determinant of mortality and reduced left ventricular ejection fraction (LVEF) independently predicting earlier mortality [8]. However, patients with DMD are often undertreated regarding heart failure therapy [8]. Historically, referrals to cardiac specialists occurred late in the disease course, contributing to poor clinical outcomes. Furthermore, the New York Heart Association classification of heart failure is based on dyspnoea and exercise intolerance, which in DMD is confounded by skeletal muscle weakness, leading to under-recognition of heart failure symptoms in non-ambulatory patients. Meta-regressions demonstrated that heart failure treatment with ACEIs and/or ARBs or beta-blockers was significantly associated with prolonged survival. Current recommendations advocate initiation of ACEI or ARB therapy in late childhood, and prophylactic treatment has been associated with a survival benefit [52, 72]. HMV may provide additional cardioprotective effects alongside pharmacotherapy [74]. Meta-regression did not demonstrate an overall survival benefit associated with glucocorticoid therapy, but the adjustment for the period is limited by the study number. Although treatment for ≥1 year has been shown to preserve ambulation and upper limb function in children and to reduce the need for scoliosis surgery [75, 76], the associated adverse effects are likely to outweigh any potential benefits in non-ambulatory adults with systolic heart failure.

This study level meta-analysis has several limitations. Assignment of birth cohorts for period analyses was occasionally challenging because patients within individual studies were born across wide time intervals. However, sensitivity analyses using alternative period classifications produced consistent findings. In some studies, follow-up may have been insufficient for death to occur, or they mainly included a paediatric population. In addition, mean age at death does not account for censoring, and only 38% of patients included in this analysis were deceased. As patients had to survive long enough to receive HMV and contemporary medical care, survival estimates in ventilated cohorts may be affected by immortal-time bias. Nevertheless, individual patient data demonstrated higher 5-year survival probabilities in more recent birth cohorts across all age groups (0 to >40 years) [16]. The study is also limited by incomplete reporting across the included studies. Insufficient data precluded more detailed analyses of body mass index, glucocorticoid type and dosing, use of mechanical insufflation–exsufflation, scoliosis surgery, and geographical variation. These factors may have contributed to the observed heterogeneity, particularly within ventilated cohorts. Future observational studies should therefore additionally report restricted mean survival time (RMST), defined as the area under the Kaplan–Meier curve, as a more robust and representative measure of average survival [77]. In addition to improvements in care over time, survival heterogeneity may partly reflect regional differences in long-term management, including timing of treatment initiation, surveillance practices, and transition from paediatric to adult care [78]. Multidisciplinary management of DMD is important and should involve specialists in respiratory and sleep medicine, cardiology, neurology, rehabilitation medicine, orthopaedic surgery, gastroenterology, nutrition, palliative care, mental health, and social care [79].

### Conclusions

Survival in DMD has improved markedly over recent decades, reflecting advances in HMV, multidisciplinary care, and cardioprotective treatment. Patients receiving HMV demonstrated substantially longer survival than non-ventilated patients, with median survival now extending into the third decade of life and continuing to improve over time. As respiratory mortality declines, cardiac and other non-respiratory complications have emerged as leading causes of death. The association between longer survival and treatment with heart failure drug therapy highlights the importance of proactive cardiac surveillance and early heart failure management.

## Supplementary Information

Below is the link to the electronic supplementary material.

Supplementary file1 (PDF 292 KB)

## Data Availability

The data presented in this study are available upon reasonable request from the corresponding author.
