# Supplementary material for: Improving survival in Duchenne muscular dystrophy across eras: a systematic review and cumulative meta-analysis
Source: J Neurol. 2026 Sep 6;273(10):564. doi: 10.1007/s00415-026-14121-4 (PMC13546358; doi:10.1007/s00415-026-14121-4)
Supplement: Supplementary file 1 [file 415_2026_14121_MOESM1_ESM.pdf]

Improving survival in Duchenne muscular dystrophy across eras: a systematic review and cumulative meta-analysis

Lara Benning[1,2], Zoe Bousraou[1,2], Bastian Gruber[1,2], Silvia Ulrich[1,2], Esther I Schwarz[1,2]

[1] Department of Pulmonology, University Hospital Zurich, Zurich Switzerland
[2] University of Zurich, Zurich, Switzerland

Online Supplement
Journal of Neurology

Correspondence to
Prof Dr med Esther Irene Schwarz
Department of Pulmonology and Sleep Disorders Centre
University Hospital Zurich
Raemistrasse 100
8091 Zurich, Switzerland
e-mail: estherirene.schwarz@usz.ch

Database search strategy:

PubMed /MEDLINE) database

up to 30.06.2026

MeSH terms:

duchenne muscular dystrophy[MeSH Terms] AND (analysis, survival[MeSH Terms] OR analyses, survival[MeSH Terms] OR life expectancies[MeSH Terms] OR mortality rate[MeSH Terms] OR mortality rates[MeSH Terms])

- ➔ 134 results

Free terms:

duchenne[Title/Abstract] AND (survival[Title/Abstract] OR mortality[Title/Abstract])

- ➔ 710 results

In total, 53 studies have been included for the final analysis. [1-53]

No search software was integrated. No authors of included studies were contacted. If only abstracts containing enough information were available and no full-texts could be accessed, these were used.

Supplementary Figures

Figure S1) Funnel plot assessing publication bias. **A)** Studies with no ventilated patients. **B)** Studies with ventilated patients.

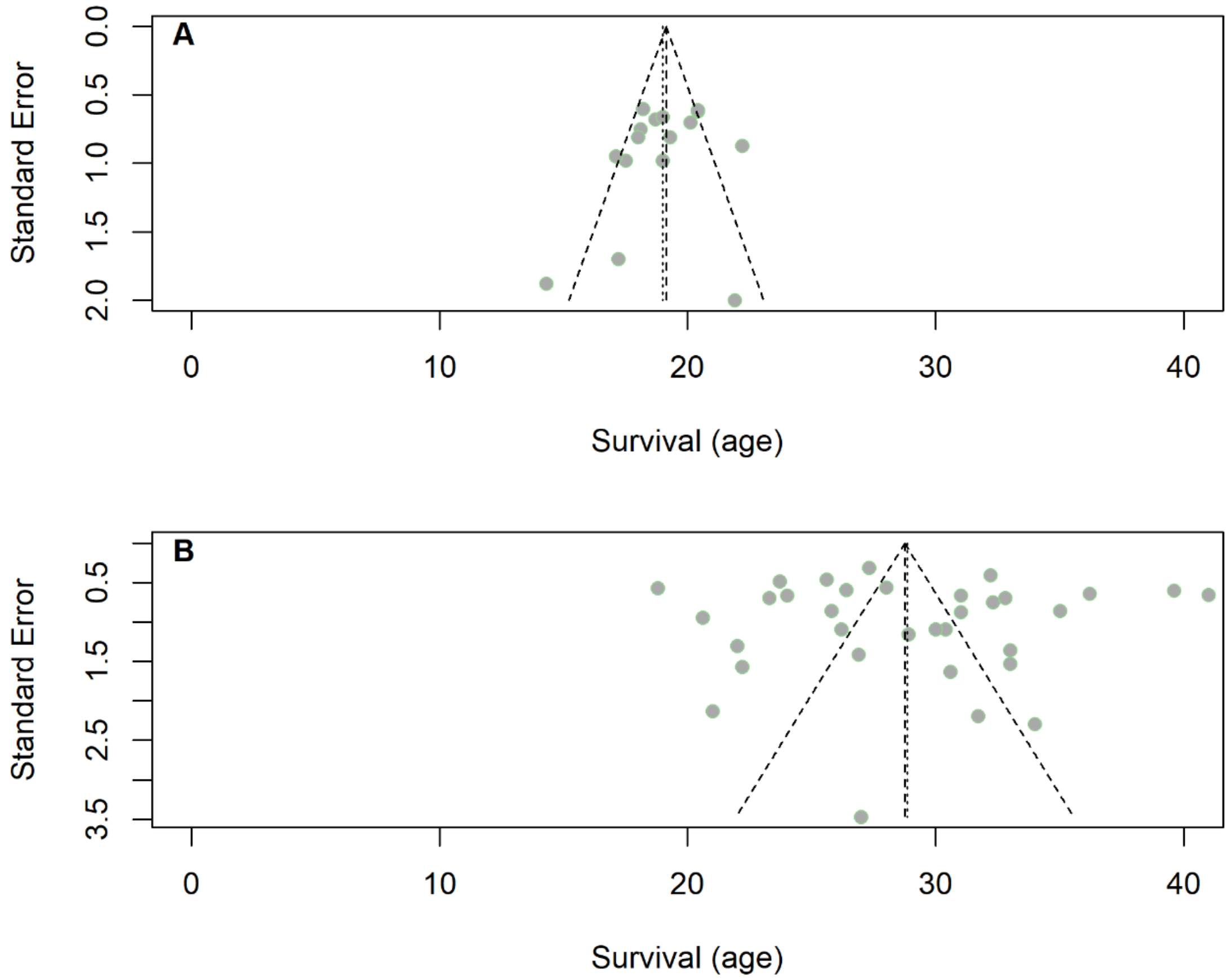

Supplementary Tables

Table S1) Characteristics of the included studies.

Study	Design	Continent (Country)	Intervention / Therapy	Year of birth	Year of inclusion	n (patients)	Deceased (%)	Ventilated (%)	Glucocorticoid intake (%)	Median survival (SE)	Age at death (mean)	NOS grading
Arshad 2024	Retrospective	Europe (UK)	Spinal fixation	NA	2000 - 2022	43	55.8	NA	NA	28.4 ± 0.86 (calculated)	NA	6
Bach 2011	Retrospective	North America (USA)	Ventilation (NIV)	NA	NA	101	44.6	100	NA	28 (Broomfield et al.) ± 0.56 (calculated)	NA	6
Benning 2026.a	Retrospective	Europe (Switzerland)	Ventilation (NIV)	1970 - 2005	1993 - 2024	55	41.8	100	NA	33 ± 1.53	26	8
Benning 2026.b	Retrospective	Europe (Switzerland)	Ventilation (IV)	1970 - 2005	1993 - 2024	25	56	100	NA	34 ± 2.29	31	8
Boland 1996	Retrospective	North America (USA)	No specific	1953 - 1983	1953 - 1993	33	51.5	NA	NA	22.7 (Broomfield et al.) ± 0.98 (calculated)	18.5	6
Boussaid 2016	Prospective	Europe (France)	Ventilation	NA	1997 - 2015	150	41	100	NA	NA	29	7
Broomfield 2023.a	Retrospective	Europe (UK)	No specific	NA	1990 - 2021	1121	30.1	50	50.2	25.6 ± 0.46	NA	6
Broomfield 2023.b	Retrospective	Europe (UK)	No specific	<1990	1990 - 2021	NA	NA	NA	NA	23.5 ± 0.74	26.9	6
Broomfield 2023.c	Retrospective	Europe (UK)	No specific	>1990	1990 - 2021	NA	NA	NA	NA	26.5 ± 0.69	29.6	7
Chataigner 1998	Retrospective	Europe (France)	Spinal fixation	NA	NA	27	37	NA	NA	NA	19	6
Cheeran 2017	Retrospective	North America (USA)	No specific	NA	2006 - 2016	43	18.6	79	56	NA	27.1	7
Connuck 2008	Ambispective	North America (USA)	No specific	NA	1990 - 2005	128	36.7	NA	NA	20.3 ± 0.5 (calculated)	NA	5
Conway 2025.a	Retrospective	North America (USA)	Prophylactic cardiac medication	1982 - 2009	NA	90	NA	28	77.8	26.4 ± 0.59 (calculated)	NA	8
Conway 2025.b	Retrospective	North America (USA)	No prophylactic cardiac medication	1982 - 2009	NA	235	NA	21	54.5	23.3 ± 0.69	NA	8
Davidson 2025.a	Retrospective	Australia (Australia)	No specific	1958 - 1969	NA	41	63	0	0	18.2 ± 0.6	NA	6
Davidson 2025.b	Retrospective	Australia (Australia)	No specific	1970 - 1979	NA	68	60	12	0	18.8 ± 0.57	NA	6
Davidson 2025.c	Retrospective	Australia (Australia)	No specific	1980 - 1989	NA	64	78	33	20	20.6 ± 0.94	NA	6
Davidson 2025.d	Retrospective	Australia (Australia)	No specific	1990 - 1999	NA	66	47	65	76	24 ± 0.66	NA	6
Dreher 2007	Retrospective	Europe (Germany)	Ventilation (NPPV)	NA	1997 - 2006	12	NA	100	NA	30.6 ± 1.63 (calculated)	NA	5
Eagle 2002.a	Retrospective	Europe (UK)	Ventilation (NIV)	NA	1967 - 2002	27	37.5	100	0	26.2 (Broomfield et al.) ± 1.09 (calculated)	25.3	7
Eagle 2002.b	Retrospective	Europe (UK)	No ventilation (no NIV)	NA	1967 - 2002	137	97.8	0	0	NA	19.3	7

Eagle 2002.c	Retrospective	Europe (UK)	No ventilation (no NIV)	NA	1960 - 1969	9	100	0	NA	14.3 (Broomfield et al.) ± 1.88 (calculated)	NA	7
Eadle 2002.d	Retrospective	Europe (UK)	No ventilation (no NIV)	NA	1970 - 1979	49	100	0	NA	18 (Broomfield et al.) ± 0.81 (calculated)	NA	7
Eagle 2002.e	Retrospective	Europe (UK)	No ventilation (no NIV)	NA	1980 - 1989	68	100	0	NA	18.7 (Broomfield et al.) ± 0.68 (calculated)	NA	7
Eagle 2002.f	Retrospective	Europe (UK)	No ventilation (no NIV)	NA	1990 - 2002	33	100	0	NA	19 (Broomfield et al.) ± 0.98 (calculated)	NA	7
Eagle 2007.a	Retrospective	Europe (UK)	HMV + spinal fixation	1970 - 1990	NA	17	NA	100	NA	30 ± 1.09 (calculated)	NA	8
Eagle 2007.b	Retrospective	Europe (UK)	HMV + no spinal fixation	1970 – 1990	NA	13	NA	100	NA	22.2 ± 1.57 (calculated)	NA	8
Eagle 2007.c	Retrospective	Europe (UK)	no HMV + no spinal fixation	1970 - 1990	NA	35	NA	0	NA	17.1 ± 0.95 (calculated)	NA	8
Fraser 2012.a	Retrospective	Europe (UK)	No specific	1970 - 1979	1987 - 2010	87	NA	NA	NA	20.2 ± 0.68	NA	5
Fraser 2012.b	Retrospective	Europe (UK)	No specific	1980 - 1989	1987 - 2010	43	NA	NA	NA	24.7 ± 0.86 (calculated)	NA	5
Gordon 2011.a	Prospective	North America (Canada)	Bisphosphonates	1963 - 2006	1963 - 2009	16	30	6	100	26.9 (Broomfield et al.) ± 1.41 (calculated)	NA	5
Gordon 2011.b	Prospective	North America (Canada)	No bisphosphonates	1963 - 2006	1963 - 2009	28	100	NA	100	20.9 (Broomfield et al.) ± 1.07 (calculated)	NA	5
Hinge 1990	Retrospective	Europe (Denmark)	Daily physiotherapy	NA	1975 - 1989	81	NA	NA	NA	22 ± 0.63 (calculated)	NA	5
Iff 2024	Retrospective	North America (USA)	Antisense oligonucleotides	1985 - 2020	1980 - 2022	579	NA	NA	NA	32.8 ± 0.23 (calculated)	NA	5
Ishikawa 2011.a	Retrospective	Asia (Japan)	No ventilation	NA	1964 - 1984	56	100	0	NA	18.1 ± 0.75 (calculated)	18.6	7
Ishikawa 2011.b	Retrospective	Asia (Japan)	Ventilation (IV)	NA	1984 - 1991	24	87.5	100	NA	28.9 ± 1.15 (calculated)	28.1	7
Ishikawa 2011.c	Retrospective	Asia (Japan)	Ventilation (NIV) + Cardiac medication	NA	1991 - 2010	88	19.4	100	8	39.6 ± 0.6 (calculated)	27.1	7
Ishikawa 2011.d	Retrospective	Asia (Japan)	No ventilation	NA	1984 - 1991	11	NA	0	NA	17.2 (Broomfield et al.) ± 1.7 (calculated)	NA	7
Ishikawa 2011.e	Retrospective	Asia (Japan)	No Ventilation	NA	1991 - 2010	8	NA	0	NA	21.9 (Broomfield et al.) ± 2 (calculated)	NA	7
Kanamori 1988	Retrospective	Asia (Japan)	No specific	NA	NA	666	NA	NA	NA	22.5 ± 0.22 (calculated)	19.5	5
Kennedy 1995.a	Retrospective	Australia (Australia)	Spinal fixation	NA	1960 - 1993	17	58.8	NA	NA	NA	18.2	6
Kennedy 1995.b	Retrospective	Australia (Australia)	No spinal fixation	NA	1960 - 1993	21	52.4	NA	NA	NA	19.5	6
Kieny 2013.a	Retrospective	Europe (France)	Ventilation	NA	1981 - 2011	77	NA	100	0	36.2 ± 0.64 (calculated)	28.3	7
Kieny 2013.b	Retrospective	Europe (France)	No ventilation	NA	1981 - 2011	42	NA	0	0	22.2 ± 0.87 (calculated)	21.8	7
Kieny 2013.c	Retrospective	Europe (France)	No specific	1955 - 1970	NA	43	NA	61	0	25.8 ± 0.86 (calculated)	NA	7

Kieny 2013.d	Retrospective	Europe (France)	No specific	1970 - 1994	NA	76	NA	83	0	41 ± 0.65 (calculated)	NA	7
Kinali 2006	Retrospective	Europe (UK)	Spinal fixation	NA	1992 - 2002	16	93.8	81	NA	NA	20.2	5
Kisel 2023	Retrospective	Europe (UK)	No specific	NA	1993 - 201	68	23.5	90	16.2	NA	26	5
Kohler 2009	Prospective	Europe (Switzerland)	No specific	NA	1999 - 2006	43	7	51	NA	35 ± 0.86 (calculated)	NA	6
Konagaya 2005.a	Retrospective	Asia (Japan)	Ventilation (IPPV)	NA	Since 1990	73	39.7	100	NA	31 ± 0.66 (calculated)	25.7	6
Konagaya 2005.b	Retrospective	Asia (Japan)	No ventilation (no IPPV)	NA	Since 1990	84	88.1	0	NA	20.4 ± 0.62 (calculated)	19.8	6
Lechner 2023	Retrospective	Europe (Switzerland)	No specific	NA	1996 - 2022	67	42	88	NA	33 ± 1.36	29.2	8
LoMauro 2021	Retrospective	Europe (Italy)	No specific	NA	2000 - 2017	132	15.2	NA	31.1	NA	19	5
Matsumura 2011.a	Retrospective	Asia (Japan)	No ventilation	NA	1977 - 1984	33	NA	0	NA	17.5 (Broomfield et al.) ± 0.98 (calculated)	18.9	5
Matsumura 2011.b	Retrospective	Asia (Japan)	No specific	NA	1984 - 1993	204	NA	NA	NA	32.2 (Broomfield et al.) ± 0.4 (calculated)	20	5
Matsumura 2011.c	Retrospective	Asia (Japan)	No specific	NA	1994 - 2003	NA	NA	NA	NA	32.8 (Broomfield et al.) ± 0.69 (calculated)	25.2	5
Matsumura 2011.d	Retrospective	Asia (Japan)	No ventilation	NA	1984 - 2010	49	NA	0	NA	19.3 (Broomfield et al.) ± 0.81 (calculated)	NA	5
Matsumura 2011.e	Retrospective	Asia (Japan)	No specific	NA	2004 - 2010	NA	NA	NA	NA	NA	31.1	5
Mochizuki 2008	Retrospective	Asia (Japan)	No specific	NA	1995 - 2007	194	21.1	NA	NA	NA	25.2	5
Nart 2024.a	Retrospective	Europe (UK)	No specific	NA	2011 - 2022	37	100	NA	62.2	NA	23.9	5
Nart 2024.b	Retrospective	Europe (UK)	No specific	NA	2011 - 2015	9	100	NA	NA	NA	20.1	5
Nart 2024.c	Retrospective	Europe (UK)	No specific	NA	2016 - 2020	16	100	NA	NA	NA	22.2	5
Nart 2024.d	Retrospective	Europe (UK)	No specific	NA	2021 - 2022	12	100	NA	NA	NA	26.7	5
Pandya 2018	Retrospective	North America (USA)	No specific	Since 1982	1982 - 2012	208	28.3	69	48.5	NA	21.5	6
Paramsothy 2022	Retrospective	North America (USA)	No specific	1982 - 1999	1982 - 2011	504	27	38	43.7	23.7 ± 0.48	19	8
Phillips 1999	Retrospective	Europe (UK)	No specific	NA	NA	19	84.2	11	NA	22 ± 1.3 (calculated)	22	6
Phillips 2001	Retrospective	Europe (UK)	No specific	NA	1986 - 1999	58	63.8	NA	NA	21.5 ± 0.74 (calculated)	20.5	5
Porcher 2021.a	Retrospective	Europe (France)	Prophylactic ACE inhibitor	NA	1986 - 2018	390	13.5	NA	27.6	NA	20	9
Porcher 2021.b	Retrospective	Europe (France)	No specific	NA	1986 - 2018	186	32.3	NA	26.9	NA	21	9

Qian 2025	Retrospective	North America (Canada)	No specific	NA	1998 - 2020	198	13.6	NA	25.8	NA	20.4	6
Rall 2012.a	Retrospective	Europe (Germany)	No specific	NA	1970 - 1980	67	83.6	NA	NA	24 ± 1.38	NA	4
Rall 2012.b	Retrospective	Europe (Germany)	Ventilation	NA	1970 - 1980	44	NA	100	NA	27 ± 3.47	NA	4
Rall 2012.c	Retrospective	Europe (Germany)	No ventilation	NA	1970 - 1980	22	NA	0	NA	19 ± 0.66	NA	4
Saito 2014.a	Retrospective	Asia (Japan)	No specific	NA	1999 - 2000	873	NA	59	NA	NA	26.7	5
Saito 2014.b	Retrospective	Asia (Japan)	No specific	NA	2000 - 2012	733	58.6	NA	NA	NA	32.4	5
San Martin 2018.a	Prospective	South America (Chile)	No specific	NA	1993 - 2002	244	62.7	NA	NA	19.1 ± 0.04	18.3	5
San Martin 2018.b	Prospective	South America (Chile)	No specific	NA	1993 - 2013	462	35.9	NA	NA	20.2 (Broomfield et al.) ± 0.26 (calculated)	18.1	5
San Martin 2018.c	Prospective	South America (Chile)	No specific	NA	2003 - 2013	218	6	NA	NA	NA	16.3	5
Schiava 2024	Retrospective	Europe (UK)	No specific	1971 - 2005	1986 - 2022	112	NA	60	47.2	NA	25	6
Soslow 2023	Prospective	North America (USA)	(Cardiac MR)	NA	2007 - 2020	78	19.2	9	59	NA	17	5
Soudon 2008.a	Prospective	Europe (Belgium)	Ventilation (IV)	NA	2002 - 2006	16	50	100	NA	NA	35.8	6
Soudon 2008.b	Prospective	Europe (Belgium)	Ventilation (NIV)	NA	2002 - 2006	26	38.5	100	NA	NA	30.4	6
Starnes 2024	Prospective	North America (USA)	(Cardiac MR)	NA	NA	179	10.9	NA	75.4	NA	17.8	6
Toussaint 2006	Prospective	Europe (Belgium)	Ventilation (MIPPV)	NA	1996 - 2005	42	26.2	100	0	31 ± 0.87 (calculated)	25	6
Trucco 2020	Retrospective	Europe (UK)	No specific	NA	2000 - 2017	270	2	7	92	NA	16.5	6
van den Bergen 2014.a	Retrospective	Europe (Netherlands)	No specific	1961 - 1974	1961 - 1998	293	33.4	NA	NA	NA	16	5
van den Bergen 2014.b	Retrospective	Europe (Netherlands)	No specific	1980 - 2006	NA	336	12.2	28	35	27.3 ± 0.31 (calculated)	19	5
van den Bergen 2014.c	Retrospective	Europe (Netherlands)	No Glucocorticoids	1980 - 2006	NA	150	17.3	NA	0	NA	19	5
Wahlgren 2022.a	Retrospective	Europe (Sweden)	No specific	1970 - 1979	1970 - 2019	26	83.9	NA	21.7	NA	30.7	6
Wahlgren 2022.b	Retrospective	Europe (Sweden)	No specific	1980 - 1989	1980 - 2019	63	63.6	NA	64.3	29.6 ± 1.48	24.8	6
Wahlgren 2022.c	Retrospective	Europe (Sweden)	No specific	1990 - 1999	1990 - 2019	37	32.7	NA	88.9	NA	20.1	5
Wahlgren 2022.d	Retrospective	Europe (Sweden)	No specific	2000 - 2009	2000 - 2019	3	3.2	NA	100	NA	14.5	5
Wahlgren 2022.e	Retrospective	Europe (Sweden)	No specific	1980 - 2009	1980 - 2019	373	34.6	NA	64.4	29.9 ± 1.05	24.4	5

Wang 2018.a	Retrospective	North America (USA)	No specific	NA	2003 - 2015	57	47	88	26.3	31.7 ± 2.19	26	6
Wang 2018.b	Retrospective	North America (USA)	No specific (LVD at age <18)	NA	2003 - 2015	18	NA	NA	NA	NA	21.1	6
Wang 2018.c	Retrospective	North America (USA)	No specific (LVD at age >18)	NA	2003 - 2015	22	NA	NA	NA	NA	33.1	6
Wang 2018.d	Retrospective	North America (USA)	No specific (LVD)	NA	2003 - 2015	40	NA	NA	22.5	NA	25.7	6
Wang 2018.e	Retrospective	North America (USA)	No specific (no LVD)	NA	2003 - 2015	17	NA	NA	35.3	NA	27.6	6
Wittlieb-Weber 2019	Prospective	North America (USA)	No specific	NA	2005 - 2018	436	6.8	NA	77	NA	21.8	6
Wittlieb-Weber 2020	Retrospective	North America (USA)	No specific	NA	2005 - 2015	408	7.1	NA	78.2	27.1 (Broomfield et al.) ± 0.28 (calculated)	20.4	7
Yasuma 1996.a	Retrospective	Asia (Japan)	No ventilation	NA	1980 - 1987	65	NA	0	NA	20.1 ± 0.7 (calculated)	NA	5
Yasuma 1996.b	Retrospective	Asia (Japan)	Ventilation (Cuirass)	NA	1987 - 1992	7	NA	100	NA	21 ± 2.13 (calculated)	NA	5
Yasuma 1996.c	Retrospective	Asia (Japan)	Ventilation (NIPPV)	NA	1992 - 1995	27	NA	100	NA	30.4 ± 1.09 (calculated)	NA	5
Yasuma 2004.a	Retrospective	Asia (Japan)	Ventilation (IPPV)	NA	1980 - 2002	56	42.8	100	NA	32.3 ± 0.75 (calculated)	NA	6
Yasuma 2004.b	Retrospective	Asia (Japan)	No Ventilation (no IPPV)	NA	1980 - 2002	85	83.5	0	NA	20.4 ± 0.61 (calculated)	NA	6

REFERENCES

1. Arshad A, Tsirikos AI (2024) Survival of patients with Duchenne muscular dystrophy who underwent spinal deformity correction. Dev Med Child Neurol 66:187-194
2. Bach JR, Martinez D (2011) Duchenne muscular dystrophy: continuous noninvasive ventilatory support prolongs survival. Respir Care 56:744-750
3. Benning L, Brunner TS, Mollet M, Bradicich M, Bousraou Z, Ulrich S, Schwarz EI (2026) Long-term outcomes of home mechanical ventilation in Duchenne Muscular Dystrophy – an observational study. Respir Med 261
4. Boland BJ, Silbert PL, Groover RV, Wollan PC, Silverstein MD (1996) Skeletal, cardiac, and smooth muscle failure in Duchenne muscular dystrophy. Pediatr Neurol 14:7-12
5. Boussaid G, Lofaso F, Santos DB, Vaugier I, Pottier S, Prigent H, Bahrami S, Orlikowski D (2016) Impact of invasive ventilation on survival when non-invasive ventilation is ineffective in patients with Duchenne muscular dystrophy: A prospective cohort. Respir Med 115:26-32
6. Broomfield J, Abrams K, Latimer N, Guglieri M, Rutherford M, Crowther M (2023) Natural history of Duchenne muscular dystrophy in the United Kingdom: A descriptive study using the Clinical Practice Research Datalink. Brain Behav 13:e3331
7. Chataigner H, Grelet V, Onimus M (1998) [Surgery of the spine in Duchenne's muscular dystrophy]. Rev Chir Orthop Reparatrice Appar Mot 84:224-230
8. Cheeran D, Khan S, Khera R, Bhatt A, Garg S, Grodin JL, Morlend R, Araj FG, Amin AA, Thibodeau JT, Das S, Drazner MH, Mammen PPA (2017) Predictors of Death in Adults With Duchenne Muscular Dystrophy-Associated Cardiomyopathy. J Am Heart Assoc 6
9. Connuck DM, Sleeper LA, Colan SD, Cox GF, Towbin JA, Lowe AM, Wilkinson JD, Orav EJ, Cuniberti L, Salbert BA, Lipshultz SE, Pediatric Cardiomyopathy Registry Study G (2008) Characteristics and outcomes of cardiomyopathy in children with Duchenne or Becker muscular dystrophy: a comparative study from the Pediatric Cardiomyopathy Registry. Am Heart J 155:998-1005
10. Conway KM, Thomas S, Neyaz T, Ciafaloni E, Mann JR, Staron-Ehlinger M, Beasley GS, Romitti PA, Mathews KD, Muscular Dystrophy Surveillance T, Research N (2025) Prophylactic Use of Cardiac Medications and Survival in Duchenne Muscular Dystrophy. Muscle Nerve 71:574-582
11. Davidson ZE, Vidmar S, Griffiths A, Howard ME, Berlowitz DJ, Jones EF, Scully T, Treanor D, Singh B, Prior D, Frost MG, Forbes R, Billich N, Adams J, Carroll K, Al Lawati T, Bourne H, Kornberg AJ, Ryan MM, Woodcock IR, Yiu EM, Cheung MMH (2025) Survival in Duchenne muscular dystrophy in Australia: a 50 year retrospective cohort study. Lancet Reg Health West Pac 58:101568
12. Dreher M, Rauter I, Storre JH, Geiseler J, Windisch W (2007) When should home mechanical ventilation be started in patients with different neuromuscular disorders? Respirology 12:749-753
13. Eagle M, Baudouin SV, Chandler C, Giddings DR, Bullock R, Bushby K (2002) Survival in Duchenne muscular dystrophy: improvements in life expectancy since 1967 and the impact of home nocturnal ventilation. Neuromuscul Disord 12:926-929

14. Eagle M, Bourke J, Bullock R, Gibson M, Mehta J, Giddings D, Straub V, Bushby K (2007) Managing Duchenne muscular dystrophy--the additive effect of spinal surgery and home nocturnal ventilation in improving survival. Neuromuscul Disord 17:470-475
15. Fraser LK, Childs AM, Miller M, Aldridge J, Manning S, McKinney PA, Parslow RC (2012) A cohort study of children and young people with progressive neuromuscular disorders: clinical and demographic profiles and changing patterns of referral for palliative care. Palliat Med 26:924-929
16. Gordon KE, Dooley JM, Sheppard KM, MacSween J, Esser MJ (2011) Impact of bisphosphonates on survival for patients with Duchenne muscular dystrophy. Pediatrics 127:e353-358
17. Hinge HH, Hein-Sorensen O (1990) [Duchenne muscular dystrophy. Therapeutic principles and status after 14 years]. Ugeskr Laeger 152:897-900
18. Iff J, Done N, Tuttle E, Zhong Y, Wei F, Darras BT, McDonald CM, Mercuri E, Muntoni F (2024) Survival among patients receiving eteplirsen for up to 8 years for the treatment of Duchenne muscular dystrophy and contextualization with natural history controls. Muscle Nerve 70:60-70
19. Ishikawa Y, Miura T, Ishikawa Y, Aoyagi T, Ogata H, Hamada S, Minami R (2011) Duchenne muscular dystrophy: survival by cardio-respiratory interventions. Neuromuscul Disord 21:47-51
20. Kanamori M (1988) [Genetic epidemiology of Duchenne muscular dystrophy in Japan]. Hokkaido Igaku Zasshi 63:851-858
21. Kennedy JD, Staples AJ, Brook PD, Parsons DW, Sutherland AD, Martin AJ, Stern LM, Foster BK (1995) Effect of spinal surgery on lung function in Duchenne muscular dystrophy. Thorax 50:1173-1178
22. Kieny P, Chollet S, Delalande P, Le Fort M, Magot A, Pereon Y, Perrouin Verbe B (2013) Evolution of life expectancy of patients with Duchenne muscular dystrophy at AFM Yolaine de Kepper centre between 1981 and 2011. Ann Phys Rehabil Med 56:443-454
23. Kinali M, Messina S, Mercuri E, Lehovsky J, Edge G, Manzur AY, Muntoni F (2006) Management of scoliosis in Duchenne muscular dystrophy: a large 10-year retrospective study. Dev Med Child Neurol 48:513-518
24. Kisel J, Ballard E, Suh ES, Hart N, Kapetanakis S, Srivastava S, Marino P, Murphy P, Steier J (2023) Cardioprotective medication in Duchenne muscular dystrophy: a single-centre cohort study. J Thorac Dis 15:812-819
25. Kohler M, Clarenbach CF, Bahler C, Brack T, Russi EW, Bloch KE (2009) Disability and survival in Duchenne muscular dystrophy. J Neurol Neurosurg Psychiatry 80:320-325
26. Konagaya M, Sakai M, Wakayama T, Kimura S, Kuru S, Yasuma F (2005) [Effect of intermittent positive pressure ventilation on life-span and causes of death in Duchenne muscular dystrophy]. Rinsho Shinkeigaku 45:643-646
27. Lechner A, Herzig JJ, Kientsch JG, Kohler M, Bloch KE, Ulrich S, Schwarz EI (2023) Cardiomyopathy as cause of death in Duchenne muscular dystrophy: a longitudinal observational study. ERJ Open Res 9

28. LoMauro A, Gandossini S, Russo A, Velardo D, Comi GP, Turconi AC, Bresolin N, Aliverti A, D'Angelo MG (2021) A Multidisciplinary Evaluation of Patients with DMD in An Italian Tertiary Care Center. J Neuromuscul Dis 8:235-249
29. Matsumura T, Saito T, Fujimura H, Shinno S, Sakoda S (2011) [A longitudinal cause-of-death analysis of patients with Duchenne muscular dystrophy]. Rinsho Shinkeigaku 51:743-750
30. Mochizuki H, Miyatake S, Suzuki M, Shigeyama T, Yatabe K, Ogata K, Tamura T, Kawai M (2008) Mental retardation and lifetime events of Duchenne muscular dystrophy in Japan. Intern Med 47:1207-1210
31. Nart L, Desikan M, Pietrusz A, Savvatis K, Quinlivan R (2024) Neurodiversity, treatment compliance and survival in adults with Duchenne muscular dystrophy: a single-centre retrospective cohort review. Neuromuscul Disord 35:13-18
32. Pandya S, James KA, Westfield C, Thomas S, Fox DJ, Ciafaloni E, Moxley RT (2018) Health profile of a cohort of adults with Duchenne muscular dystrophy. Muscle Nerve 58:219-223
33. Paramsothy P, Wang Y, Cai B, Conway KM, Johnson NE, Pandya S, Ciafaloni E, Mathews KD, Romitti PA, Howard JF, Jr., Riley C (2022) Selected clinical and demographic factors and all-cause mortality among individuals with Duchenne muscular dystrophy in the Muscular Dystrophy Surveillance, Tracking, and Research Network. Neuromuscul Disord 32:468-476
34. Phillips MF, Quinlivan RC, Edwards RH, Calverley PM (2001) Changes in spirometry over time as a prognostic marker in patients with Duchenne muscular dystrophy. Am J Respir Crit Care Med 164:2191-2194
35. Phillips MF, Smith PE, Carroll N, Edwards RH, Calverley PM (1999) Nocturnal oxygenation and prognosis in Duchenne muscular dystrophy. Am J Respir Crit Care Med 160:198-202
36. Porcher R, Desguerre I, Amthor H, Chabrol B, Audic F, Rivier F, Isapof A, Tiffreau V, Campana-Salort E, Leturcq F, Tuffery-Giraud S, Ben Yaou R, Annane D, Amedro P, Barnerias C, Becane HM, Behin A, Bonnet D, Bassez G, Cossee M, de La Villeon G, Delcourte C, Fayssoil A, Fontaine B, Godart F, Guillaumont S, Jaillette E, Laforet P, Leonard-Louis S, Lofaso F, Mayer M, Morales RJ, Meune C, Orlikowski D, Ovaert C, Prigent H, Saadi M, Sochala M, Tard C, Vaksmann G, Walther-Louvier U, Eymard B, Stojkovic T, Ravaud P, Duboc D, Wahbi K (2021) Association between prophylactic angiotensin-converting enzyme inhibitors and overall survival in Duchenne muscular dystrophy-analysis of registry data. Eur Heart J 42:1976-1984
37. Qian C, Klimchak AC, Szabo SM, Gooch KL, Dragan R, Prior HJ, Mah JK (2025) Longitudinal Outcomes Among Patients With Duchenne Muscular Dystrophy: A Canadian Retrospective Population-Based Study. Muscle Nerve 71:955-962
38. Rall S, Grimm T (2012) Survival in Duchenne muscular dystrophy. Acta Myol 31:117-120
39. Saito T, Tatara K, Kawai M (2014) [Changes in clinical condition and causes of death of inpatients with Duchenne muscular dystrophy in Japan from 1999 to 2012]. Rinsho Shinkeigaku 54:783-790
40. San Martin PP, Solis FF, Cavada Ch G (2018) Survival of patients with Duchenne muscular dystrophy. Rev Chil Pediatr 89:477-483

41. Schiava M, Lofra RM, Bourke JP, Diaz-Manera J, James MK, Elseed MA, Malinova M, Michel-Sodhi J, Moat D, Ghimenton E, McCallum M, Diaz CFB, Mayhew A, Wong K, Richardson M, Tasca G, Eglon G, Eagle M, Turner C, Heslop E, Straub V, Bettolo CM, Guglieri M (2024) Functional abilities, respiratory and cardiac function in a large cohort of adults with Duchenne muscular dystrophy treated with glucocorticoids. Eur J Neurol 31:e16267
42. Soslow JH, Xu M, Slaughter JC, Crum K, Kaslow JA, George-Durrett K, Raucci FJ, Jr., Wilkinson JD, Cripe LH, Hor KN, Spurney CF, Markham LW (2023) Cardiovascular Measures of All-Cause Mortality in Duchenne Muscular Dystrophy. Circ Heart Fail 16:e010040
43. Soudon P, Steens M, Toussaint M (2008) A comparison of invasive versus noninvasive full-time mechanical ventilation in Duchenne muscular dystrophy. Chron Respir Dis 5:87-93
44. Starnes JR, Weiner JG, George-Durrett K, Crum K, Henderson CC, Campbell MJ, Gambetta K, Hor KN, Husain N, Li JS, Raucci FJ, Jr., Soriano BD, Spurney CF, Markham LW, Soslow JH (2024) Boys With Duchenne Muscular Dystrophy Have Diastolic Dysfunction Based on CMR. Circ Cardiovasc Imaging 17:e017287
45. Toussaint M, Steens M, Wasteels G, Soudon P (2006) Diurnal ventilation via mouthpiece: survival in end-stage Duchenne patients. Eur Respir J 28:549-555
46. Trucco F, Domingos JP, Tay CG, Ridout D, Maresh K, Munot P, Sarkozy A, Robb S, Quinlivan R, Riley M, Burch M, Fenton M, Wallis C, Chan E, Abel F, Manzur AY, Muntoni F (2020) Cardiorespiratory Progression Over 5 Years and Role of Corticosteroids in Duchenne Muscular Dystrophy: A Single-Site Retrospective Longitudinal Study. Chest 158:1606-1616
47. van den Bergen JC, Ginjaar HB, van Essen AJ, Pangalila R, de Groot IJ, Wijkstra PJ, Zijnen MP, Cobben NA, Kampelmacher MJ, Wokke BH, de Coo IF, Fock JM, Horemans AM, van Tol M, Vroom E, Rijlaarsdam ME, Straathof CS, Niks EH, Verschuuren JJ (2014) Forty-Five Years of Duchenne Muscular Dystrophy in The Netherlands. J Neuromuscul Dis 1:99-109
48. Wahlgren L, Kroksmark AK, Tulinius M, Sofou K (2022) One in five patients with Duchenne muscular dystrophy dies from other causes than cardiac or respiratory failure. Eur J Epidemiol 37:147-156
49. Wang M, Birnkrant DJ, Super DM, Jacobs IB, Bahler RC (2018) Progressive left ventricular dysfunction and long-term outcomes in patients with Duchenne muscular dystrophy receiving cardiopulmonary therapies. Open Heart 5:e000783
50. Wittlieb-Weber CA, Knecht KR, Villa CR, Cunningham C, Conway J, Bock MJ, Gambetta KE, Lal AK, Schumacher KR, Law SP, Deshpande SR, West SC, Friedland-Little JM, Lytrivi ID, McCulloch MA, Butts RJ, Weber DR, Johnson JN (2020) Risk Factors for Cardiac and Non-cardiac Causes of Death in Males with Duchenne Muscular Dystrophy. Pediatr Cardiol 41:764-771
51. Wittlieb-Weber CA, Villa CR, Conway J, Bock MJ, Gambetta KE, Johnson JN, Lal AK, Schumacher KR, Law SP, Deshpande SR, West SC, Friedland-Little JM, Lytrivi ID, McCulloch MA, Butts RJ, Weber DR, Knecht KR (2019) Use of advanced heart failure therapies in Duchenne muscular dystrophy. Prog Pediatr Cardiol 53:11-14
52. Yasuma F, Konagaya M, Sakai M, Kuru S, Kawamura T (2004) A new lease on life for patients with Duchenne muscular dystrophy in Japan. Am J Med 117:363

53. Yasuma F, Sakai M, Matsuoka Y (1996) Effects of noninvasive ventilation on survival in patients with Duchenne's muscular dystrophy. Chest 109:590
